# The Effects of Anti-LAP Monoclonal Antibody Down-regulation of CD4+LAP+ T Cells on Allogeneic Corneal Transplantation in Mice

**DOI:** 10.1038/s41598-018-26235-5

**Published:** 2018-05-22

**Authors:** Shang Li, Hongshuang Lu, Ruti Sella, Wei Zhang, Hongwei Dong, Chungang Guo, Natalie A. Afshari, Zhiqiang Pan, Ying Jie

**Affiliations:** 1 Beijing Tongren Eye Center, Beijing Tongren Hospital, Capital Medical University, Beijing Ophthalmic and Visual Science Key Lab, Beijing, China; 2 Department of Ophthalmology, Beijing YouAn Hospital, Capital Medical University, Beijing, China; 3 Shiley Eye Institute, Department of Ophthalmology, University of California San Diego, La Jolla, CA USA

## Abstract

CD4+latency-associated peptide (LAP)+ T cells are a newly discovered T cell subset with suppressive function on immune responses. In this study, we investigate the role of CD4+LAP+ T cells on mice corneal allograft survival by down-regulating their expression using anti-LAP mAb. We show that a blockage of LAP leads to a decrease in the percentage of T cells expressing CD4+Foxp3+, CD4+GARP+, CD4+LAP+ and CD4+IL-10+ in the lymph nodes and spleens of mice undergoing orthotopic penetrating transplantation of corneal allograft, without affecting corneal graft survival. In addition, higher percentages of CD4+IFN-γ+ and CD4+IL-17A+ T cells in the lymph nodes and spleens, as well as TNF, IFN-γ, IL-17A and IL-6 levels in the aqueous humor, significantly increase in mice with rejected corneal grafts. The expression of TGF-β1 decreases in corneal grafts during corneal rejection period. It is therefore possible that anti-LAP mAb can down-regulate the regulatory T cell subsets with its immunosuppressive effects. The rejection of corneal grafts seems to mainly be associated with the up-regulation of Th1 and Th17 cell subsets in peripheral lymph nodes.

## Introduction

Corneal diseases are the second main cause of blindness in the world^1^. Penetrating keratoplasty is the most common form of solid tissue transplantation^2^. Although the one year graft survival for corneal allotransplantation in low-risk corneal transplants is more than 90%, immune rejection is still the major cause of graft failure^3^. At present, the most important mechanism to preserve the immune privilege of the cornea is suppression of the host immunorejection responses by activated regulatory T cells (Tregs)^4^. In recent years, several studies have reported that CD4+Foxp3+ T cells are crucial for the protection of corneal grafts from rejection^5–7^.

It is well known that CD4+Foxp3+ T cells can be mainly divided into natural Tregs (nTregs) and inducible Tregs (iTregs). These cells share some common features including expression of Foxp3 and secretion of inhibitory cytokine IL-10 and TGF-β^8^. Foxp3 expression, however, was also found in activated effector T cells (Tresp)^9^. Stockis *et al*. found that glycoprotein A repetitions predominant (GARP) and latency-associated peptide (LAP) were expressed on the surface of nTregs simultaneously, but not on Tresp^10^. Therefore, GARP and LAP are considered to be new specific markers of activated Tregs.

LAP and TGF-β form inactive complexes on the surfaces of CD4+CD25+ T cells, known as latent TGF-β complexes^11^. GARP is a latent TGF-β binding protein that functions by regulating the bioavailability and activation of TGF-β, mediating CD4+CD25+ T cells’ suppressive functions^12,13^. Several studies have demonstrated that CD4+LAP+ T cells suppress diseases such as colitis, lupus, atherosclerosis and experimental autoimmune encephalomyelitis (EAE) in animal models^14–17^. Hence, we conducted this study to explore the role of CD4+LAP+ T cells in mice after allogeneic corneal transplantation. Furthermore, we used anti-LAP mAb to assess whether it would aggravate corneal immune rejection response.

## Materials and Methods

### Animals

A total of 190 male mice, 69 wild-type C57BL/6 (H-2b) and 121 BALB/c (H-2d) mice, all six to eight weeks old, were obtained from the Beijing Wei Tong Li Hua Laboratory Animal Co., Ltd (Beijing, China). Right eye corneas, harvested from the eyes of C57BL/6 (H-2b) mice, served as allogeneic grafts for 69 of the BALB/c (H-2d) mice. Twenty-six right eye corneas from the eyes of BALB/c mice were used as syngeneic grafts. All mice were held and treated in a monitored pathogen-free environment. The experimental protocols were approved by the Animal Care and Research Committee of Capital Medical University, and all animals were treated in accordance with the Association for Research in Vision and Ophthalmology (ARVO) Statement for the Use of Animals in Ophthalmic and Vision Research.

### Corneal transplantation

Standard protocol for murine orthotopic corneal transplantation was implemented, and anesthesia with 4‰ pentobarbital sodium intra-peritoneal injection was utilized, as described previously^18^. Briefly, 2-mm-diameter donor corneas were placed in the same sized recipient bed with eight interrupted sutures (11-0 nylon; Yake). A topical antibiotic (0.3% tobramycin eye ointment) was applied in the conjunctival sac immediately after surgery, and the eyelids were sutured using 11-0 nylon sutures (Johnson). The eyelid sutures were removed three days after surgery, and the corneal sutures were removed seven days after surgery.

### Experimental design and medical interventions

The allograft recipients (C57BL/6-to-BALB/c) (n = 69) were randomly divided into the anti-LAP monoclonal antibody treated group (n = 26), in which mice were intraperitoneally injected with 50 μg of anti-LAP mAb (clone TW7-20B9, Biolegend, USA), and the isotype IgG1 (Biolegend, USA) treated group (n = 43), in which mice were intraperitoneally injected with mouse IgG1 antibody (Biolegend, USA) dissolved in 200 μl of PBS. Treatment was initiated on the first postoperative day and administered for five consecutive days. The isotype IgG1 treated group, was further stratified at the third post-operative week to a group of rejected grafts (“rejectors”) and a group of surviving grafts (“non-rejectors”). Only the non-rejected grafts of the anti-LAP treated mice were further analyzed. Syngeneic grafts (BALB/c-to-BALB/c) were performed in control animals (n = 26) that did not receive anti-LAP treatment.

### Assessment of the Grafts

All recipient mice were examined under a slit lamp biomicroscope three times a week after surgery. Mice with intra-operative hemorrhage, or post operative complications including signs of infection, synechiae formation or the development of cataract, were excluded from analysis. Digital photographs of the cornea were taken using a Nikon D90 camera attached to the slit lamp biomicroscope. Graft survival was defined according to a previously reported scoring system: 0, clear graft; 1+, minimal superficial nonstromal opacity; 2+, minimal deep stromal opacity with pupil margin and iris vessels visible; 3+, moderate deep stromal opacity with only the pupil margin visible; 4+, intense deep stromal opacity with the anterior chamber visible; and 5+, maximum stromal opacity with total obscuration of the anterior chamber^19^. Grafts with an opacity score of >2+ at day 7 post transplantation, and no improvement for at least one consecutive week following suture removal, were considered as immune-rejected.

### Histopathology

Five mice from each group were euthanized by cervical dislocation 21 days after surgery, and their eyeballs were enucleated and fixed in 10% formaldehyde solution and embedded in paraffin wax. The embedded corneal tissue was cut into sagittal sections of 5 µm thickness and stained with hematoxylin eosin. The stained slides were observed under a light microscope (Nikon ECLIPSE 80i, Japan). Images were acquired at x200 magnification.

### Flow cytometric analysis

Ipsilateral draining submandibular lymph nodes, cervical lymph nodes and spleens were harvested from five mice from each group before surgery. The single cell suspension from the lymph nodes and spleens was centrifuged and the final cell concentration was adjusted to 2 × 10^7^/ml. Each sample was then divided into two parts, and were analyzed at weekly intervals up to 4 weeks after surgery The 100 ul cell suspension was taken to the bottom of the flow tube and incubated with the anti-mouse CD4-FITC, anti-mouse GARP APC and anti-mouse LAP PerCP-Cy5.5 (eBioscience, CA, USA) at 4 °C for at least 30 minutes in the dark. And then washed, fixed, permeabilized and stained with anti-mouse Foxp3-PE for another 30 min at 4 °C in the dark. The other cell samples were re-suspended in RPMI 1640 media supplemented with 10% FBS, antibiotics, and L-glutamine and stimulated with 50 ng/ml PMA and 500 ng/ml ionomycin for 6 h, the last 2 h with the addition of GolgiPlug (BD Biosciences, CA, USA) at 37 °C. Samples were then incubated with anti-mouse CD4-FITC for 30 min at 4 °C, fixed with fixation/permeabilization solution, and stained with anti-mouse IL-10 PE, anti-mouse IFN-γ APC, anti-mouse IL-4 PE-Cy7, and anti-mouse IL-17A PerCP-Cy5.5 for another 30 min at 4 °C in the dark. Finally, the cells were re-suspended with the flow cytometry staining buffer, and analyzed using a BD FACS LSRII flow cytometer (BD Biosciences, CA, USA) and Cell Quest software.

### CBA

The aqueous humor samples were collected using a microinjector (Hamilton 1710 RN, SYR 100ul, Swiss). The samples were centrifuged at 4 °C for 3 min and then stored at −80 °C. Cytokines in aqueous humor samples were measured with BD CBA Mouse Th1/Th2/Th17 Cytokine Kit (BD Bioscience, CA, USA). The kit was used for the simultaneous detection of mouse interleukin-2 (IL-2), interleukin-4 (IL-4), interleukin-6 (IL-6), interferon-γ (IFN-γ), tumor necrosis factor (TNF), interleukin-17A (IL-17A), and interleukin-10 (IL-10) in a single sample. This array kit provided a mixture of seven capture beads with distinct fluorescent intensities that have been coated with capture antibodies specific for each cytokine. The operations were performed according to the manufacturer’s instruction. Dilution of the sample by the desired dilution factor (1:10) using the appropriate volume of Assay Diluent was performed. Subsequently, 50 μL of the mixed captured beads, 50 μL of the standard dilutions, and 50 μL of phycoerythrin (PE) detection reagent were added consecutively to each assay tube and incubated for 2 h at room temperature in the dark. The samples were washed with 1 mL of wash buffer (200 g) for 5 min and centrifuged. The bead pellet was re-suspended in 300 μL buffer after discarding the supernatant. Samples were measured on the BD FACS LSRII Flow Cytometer and analyzed by FCAP ArrayTM Software (BD Bioscience). The theoretical limits of detection were 0.1 pg/mL for IL-2, 0.03 pg/mL for IL-4, 1.4 pg/mL for IL-6, 0.5 pg/mL for IFN-γ, 0.9 pg/mL for TNF, 0.8 pg/mL for IL-17A, and 16.8 pg/mL for IL-10.

### RT-PCR of TGF-β1 in the graft

Corneal grafts of five recipient mice in each group were separated from the eyeballs, and immediately frozen into liquid nitrogen and stored at −80 °C. Total RNA was extracted from corneal samples using Trizol reagent (Invitrogen, USA) according to the manufacturer’s instructions. Reverse transcription of mRNA to cDNA was performed using RevertAid First Strand cDNA Synthesis Kit (Thermo, USA). Quantitative real-time polymerase chain reaction (PCR) was performed with the use of an ABI PRISM 7500 sequence- detection system (ABI, USA). The TGF-β1 sequence of forward and reverse primer was 5′ATACCAACTATTGCTTCAGCTCCACAG3′ and 5′GTACTGTGTGTCCAGGCTCCAAATAT3′, respectively. GAPDH was used as internal control. The threshold (CT value) of TGF-β1 was determined by normalizing to the GAPDH expression measured from the same sample in order to calculate a fold-change in value using the 2−ΔΔCT method.

### Statistical analysis

Statistical analysis was performed using SPSS (21.0, SPSS Inc., IBM Corporation, USA). All data were recorded as mean ± standard deviations. Graft survival was evaluated by generating Kaplan–Meier survival curves with group comparisons made by a log-rank test. All other data were compared with one-way analysis of variance (ANOVA). Comparisons between groups were tested by least significant difference (LSD) test. P-values < 0.05 were considered statistically significant. All photographs were processed using GraphPad Prism 5.0 software (GraphPad Software, USA).

## Results

### Corneal graft survival

**Figure 1:**
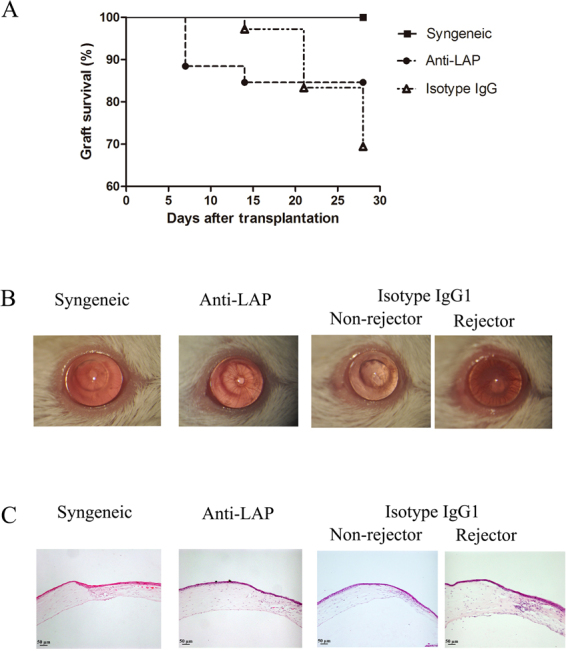
Analysis of corneal survival and transparency following transplantation. (**A**) Kaplan-Meier survival curve: there was no significant difference in survival between allografts treated with anti-LAP mAb compared with IgG1 recipients (χ^2^ = 1.276, P = 0.259). (**B**) Slit-lamp biomicroscopy: representative photomicrographs of accepted or rejected grafts were captured on day 21 following transplantation (magnifcation, x16). (**C**) Histopathology: hematoxylin and eosin staining was performed in syngeneic, anti-LAP and isotype IgG1 group on day 21 post surgery (magnifcation, x10).

Syngeneic grafts in all recipients remained transparent throughout the four-week observation period. Allogeneic grafts showed rejection signs as early as seven days after surgery in the anti-LAP treated recipients. The mice treated with isotype IgG1 showed earliest rejection signs 14 days after surgery. However, 22/26 of the allografts in the anti-LAP treated group survived (84.6% survival rate), whereas 30/43 in the isotype IgG1 treated group survived (69.8% survival rate) during the observation period. There was no significant statistical difference in survival rate of allografts between the anti-LAP and the IgG1 treated mice (χ^2^ = 1.276, P = 0.259) (Fig. 1A). The non-rejectors in the IgG1-treated mice showed a transparent corneal stroma, as seen in the syngeneic grafts. The rejectors showed severe opacity, edema, and neovascularization of the stroma (Fig. 1B). Histopathological staining of the grafts 21 days after corneal transplantation showed normal corneal thickness and few inflammatory cells infiltrating the edge of the recipient bed in the non-rejectors of the anti-LAP treated mice, similar to what was seen in the syngeneic grafts. In the rejectors of the IgG1 control mice, corneal stroma was thickened with numerous infiltrating inflammatory cells and corneal neovascularization (Fig. 1C).

### Effect of anti-LAP mAb on the CD4+ T cells in secondary lymphoid organs

**Figure 2:**
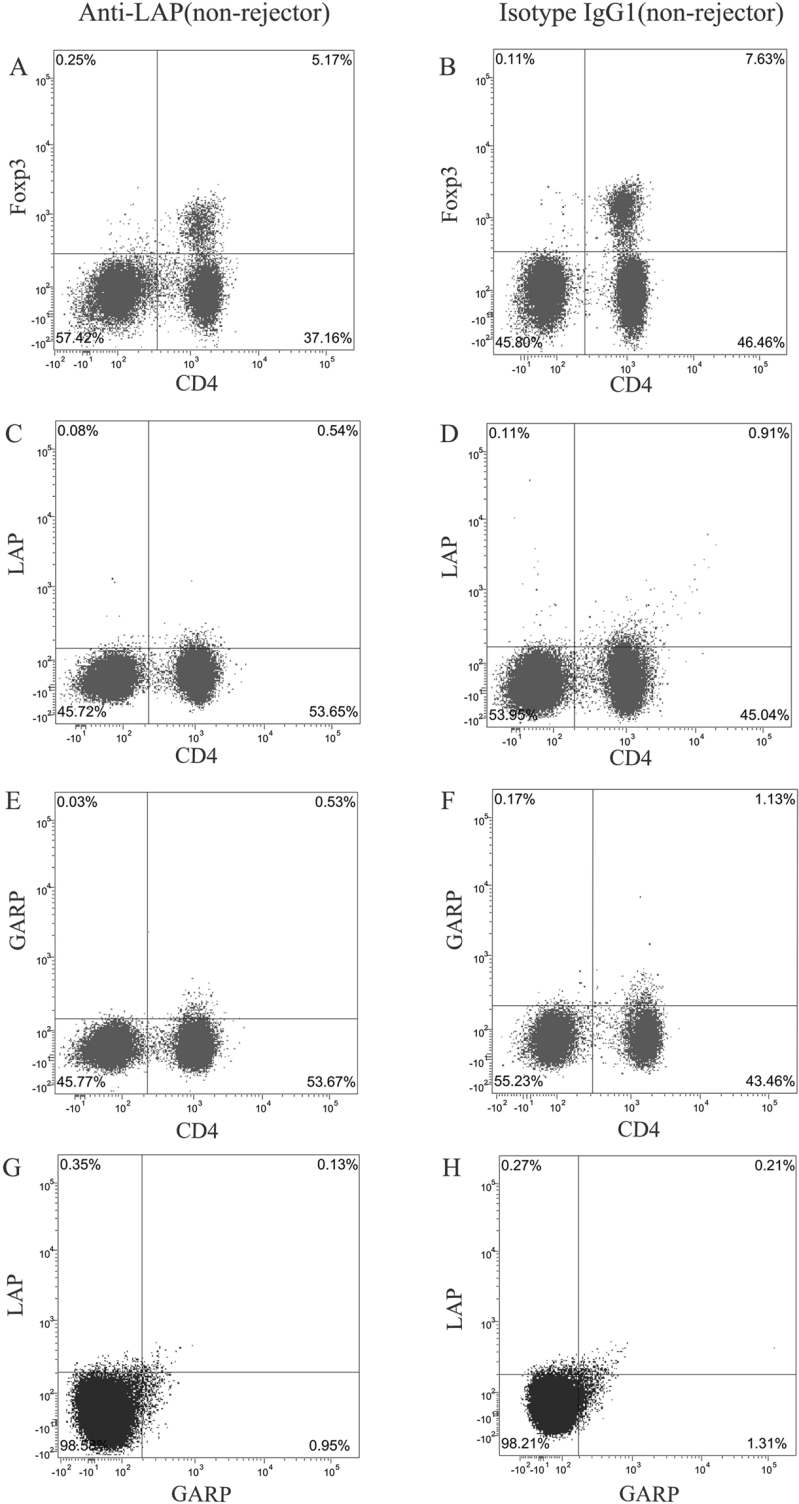
Gating strategy is represented for major cell type assessed using flow cytometry. Representative data of CD4+Foxp3+ T cells, CD4+GARP+ T cells, CD4+LAP+ T cells, CD4+GARP+LAP+ T cells between anti-LAP group and the non-rejectors of isotype IgG group in the lymph nodes at the 3^st^ week after corneal transplantation (**A~H**).

**Figure 3:**
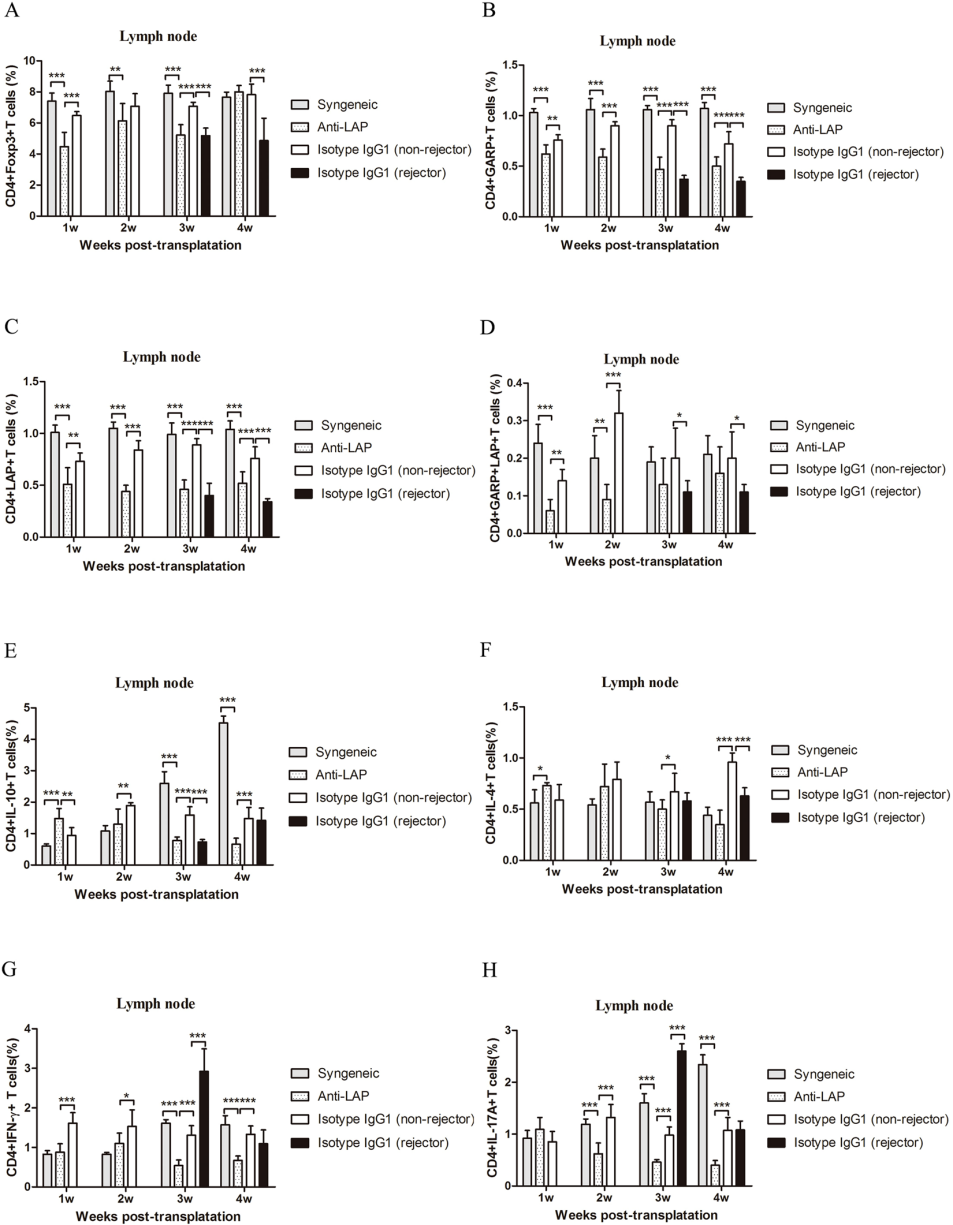
Assays of draining lymph nodes (LN) for T cells after corneal transplantation. (**A**–**H**) The numbers of CD4+Foxp3+ T cells, CD4+GARP+ T cells, CD4+LAP+ T cells, CD4+GARP+LAP+ T cells, CD4+IL-10+ T cells, CD4+IFN-γ+ T, CD4+IL-4+ T cells and CD4+IL-17A+ T cells from syngeneic mice and allogeneic mice treated with anti-LAP or isotype IgG1 were analyzed by flow cytometry. n = 5 in each group. The data are presented as the mean + SEM, *P < 0.05,**P < 0.01, ***P < 0.001.

**Figure 4:**
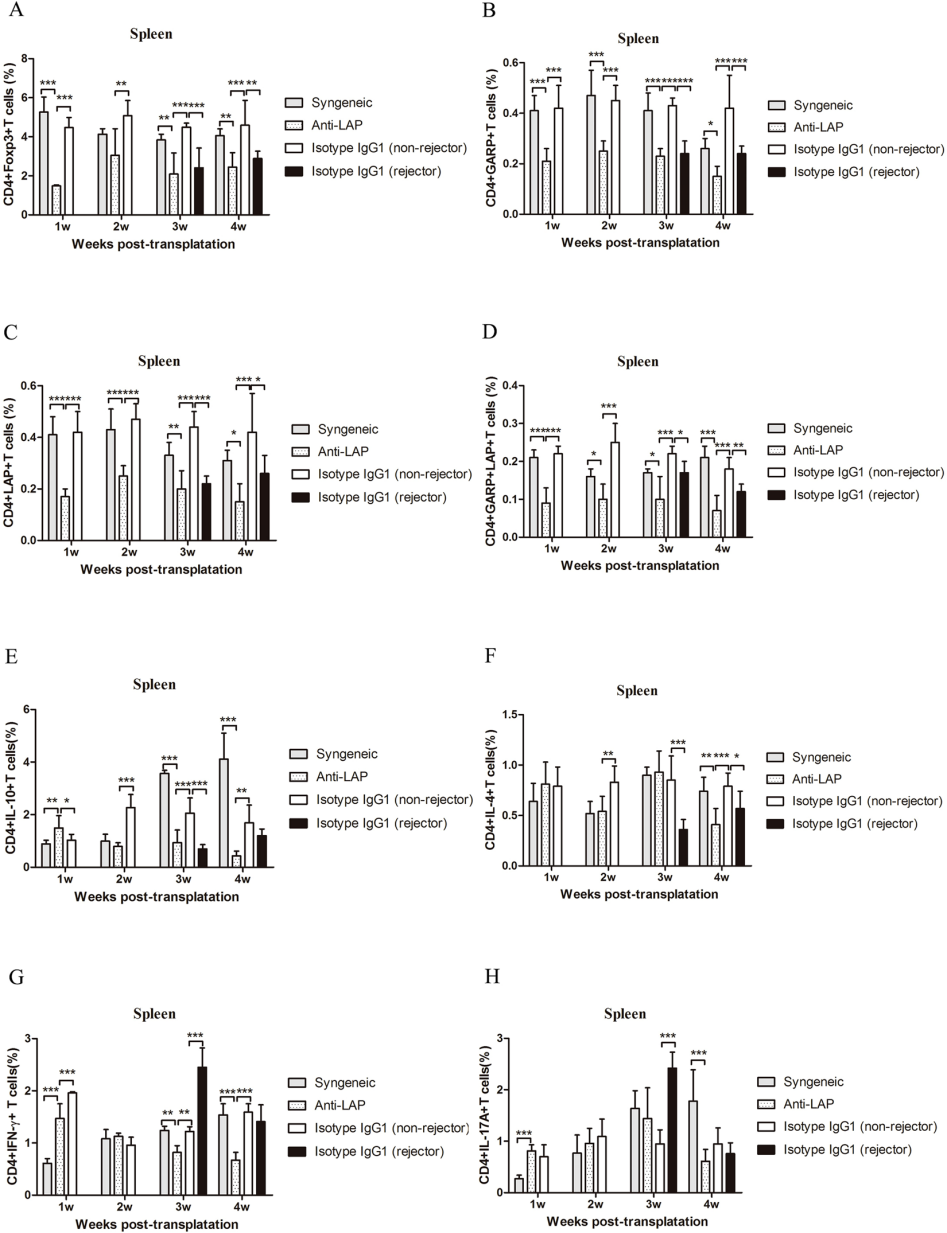
Assays of spleen (SP) for T cells after corneal transplantation. (**A**–**H**) The numbers of CD4+Foxp3+ T cells, CD4+GARP+ T cells, CD4+LAP+ T cells, CD4+GARP+LAP+ T cells, CD4+IL-10+ T cells, CD4+IFN-γ+ T, CD4+IL-4+ T cells and CD4+IL-17A+ T cells from syngeneic mice and allogeneic mice treated with anti-LAP or isotype IgG1 were analyzed by flow cytometry. n = 5 in each group. The data are presented as the mean + SEM, *P < 0.05,**P < 0.01, ***P < 0.001.

Figure 2 presents the flow cytometry results of the anti-LAP group and the non-rejectors of the isotype IgG1 treated group. Figures 3 and 4 demonstrate the CD4+ T cells expression in the secondary lymph organs, comparing the expression of Foxp3, GARP, LAP, IL-10, IFN-γ, IL-4, IL-17A, CD4+ T cells as analyzed by flow cytometry in the Syngeneic group, the non-rejector anti-LAP group, and the two IgG1 groups within 4 weeks after corneal transplantation. The percentage of CD4+Foxp3+ T cells, CD4+GARP+ T cells, CD4+LAP+ T cells and CD4+GARP+LAP+ T cells in the anti-LAP treated group was significantly lower in comparison with the non-rejectors of the isotype IgG1 treated group in the lymph nodes and spleens within the first 4 weeks after surgery (Compared with IgG1 Foxp3: P_w1 LN_ < 0.001, P_w3 LN_ < 0.001, P_w1,3,4 SP_ < 0.001, P_w2 SP_ = 0.004, GARP: P_w1 LN_ = 0.004, P_w2,3,4 LN_ < 0.001, P_w1~4 SP_ < 0.001, LAP: P_w1 LN_ = 0.007, P_w2,3,4 LN_ < 0.001, P_w1~4 SP_ < 0.001). The percentage of CD4+Foxp3+ T cells, CD4+GARP+ T cells, CD4+LAP+ T cells and CD4+GARP+LAP+ T cells in the non-rejectors was significantly higher in comparison to the rejectors in the IgG1 treated group (Foxp3: P_w3,4 LN_ < 0.001, P_w3 SP_ < 0.001, P_w4 SP_ = 0.003, GARP: P_w3,4 LN/SP_ < 0.001, LAP: P_w3,4 LN_ < 0.001, P_w3 SP_ < 0.001, P_w4 SP_ = 0.035). There was, however, no difference in the percentage of CD4+Foxp3+ T cells, CD4+GARP+ T cells, CD4+LAP+ T cells and CD4+GARP+LAP+ T cells between the non-rejectors in the anti-LAP treated mice group and rejectors in the IgG1 mice group at the third and fourth week after surgery (P > 0.05) (Figs 3A~D and 4A~D).

The percentage of CD4+IL-10+ T cells in the anti-LAP treated group was lower than those in the non-rejectors of the IgG1 treated group from the second to fourth week after surgery (P_w2 LN_ = 0.008, P_w3,4 LN_ < 0.001, P_w2 SP_ < 0.001, P_w3 SP_ = 0.002, P_w4 SP_ = 0.014) (Figs 3E and 4E). The percentage of CD4+IFN-γ+ T cells in the anti-LAP treated mice was lower than those in the non-rejectors and rejectors of the IgG1 treated mice within the first 4 weeks after surgery (Non-rejectors: P_w1,3,4 LN_ < 0.001, P_w2 LN_ = 0.034, P_w1,4 SP_ < 0.001, P_w3 SP_ = 0.007) (Rejectors: P_w3 LN_ < 0.001, P_w4 LN_ = 0.016, P_w3,4 SP_ < 0.001) (Figs 3G and 4G). The percentage of CD4+IL-17A+ T cells in the anti-LAP treated group was similar to those in the non-rejectors of the IgG1 treated group in the spleens, and lower than those in the lymph nodes from the second to fourth week after surgery (P < 0.001) (Figs 3H and 4H). Furthermore, the percentage of CD4+IFN-γ+ T cells and CD4+IL-17A+ T cells in the rejectors of IgG1 treated mice were significantly higher than those in the non-rejectors of the IgG1 treated mice in the lymph nodes and spleens at the third week after surgery (P < 0.001).

### Changes of related cytokines level in the aqueous humor

**Figure 5:**
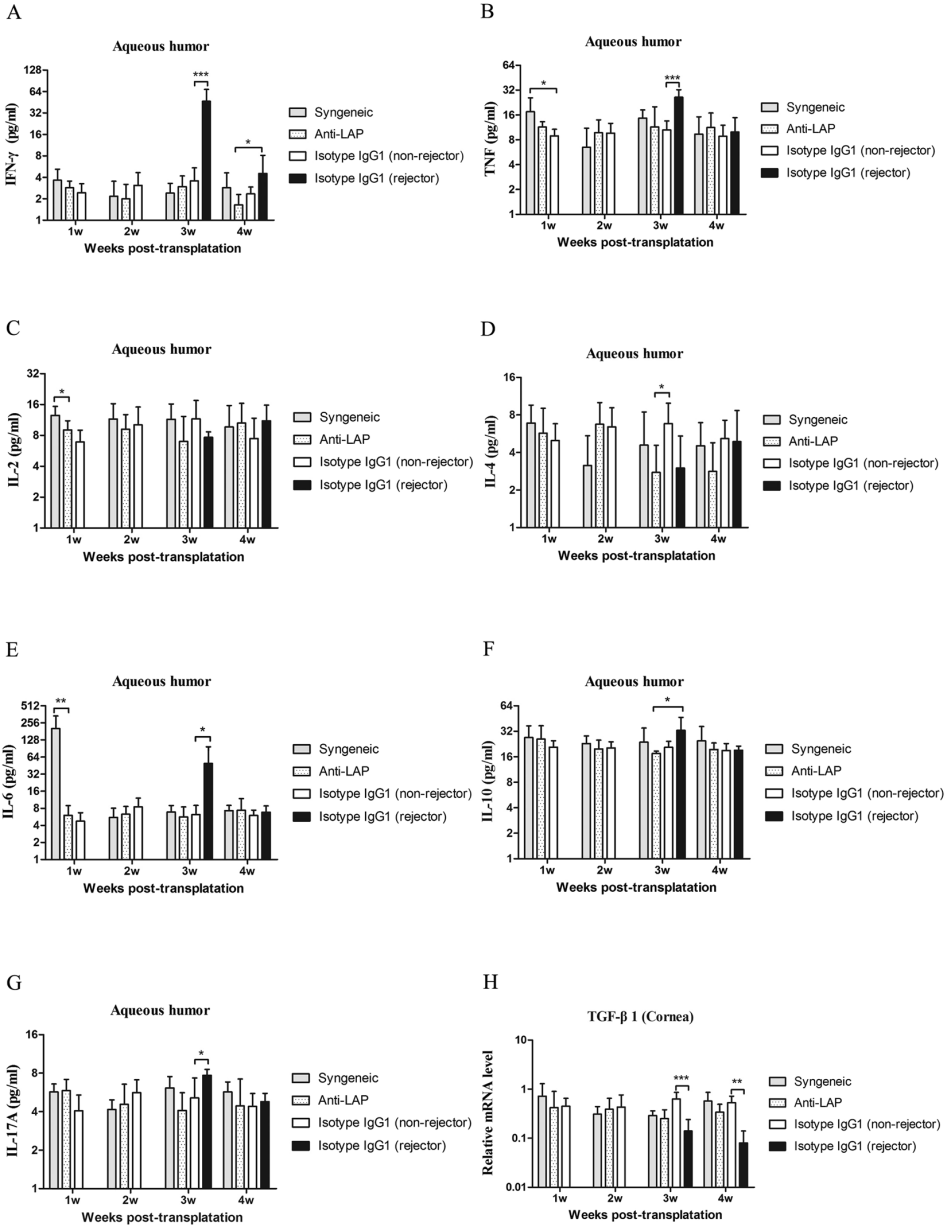
A comparison of cytokines in the aqueous humor and TGF-β1 mRNA in the corneal graft. (**A**–**G**) The concentration of IFN-γ, TNF, IL-2, IL-4, IL-6, IL-10 and IL-17A among the syngeneic group, the anti-LAP group, the isotype IgG1 group were analyzed by CBA after corneal transplantation. (**H**) The mRNA expression of TGF-β1 among groups were analyzed by RT-PCR. n = 5 in each group. The data are presented as the mean + SEM, *P < 0.05,**P < 0.01, ***P < 0.001.

Figure 5A~G demonstrates that the concentrations of IFN-γ, TNF, IL-2, IL-4, IL-6, IL-10 and IL-17A in the aqueous humor in the anti-LAP treated mice were not statistically different compared with those in the non-rejectors of the IgG1 treated group within the first 4 weeks postoperatively (P > 0.05). Moreover, there was no significant difference in the concentrations of IL-4 and IL-10 between the non-rejectors and rejectors in the IgG1 treated mice after allogeneic corneal transplantation (IL-4: P_w3_ = 0.055, P_w4_ = 0.870; IL-10: P_w3_ = 0.053, P_w4_ = 0.975)(Figs 5D and 5F). However, the concentrations of IFN-γ, TNF and IL-17A in the rejectors of the IgG1 treated group were significantly higher than those in the non-rejected mice in all three groups of syngeneic, anti-LAP treated and isotype IgG1 treated group at the third week after surgery (P _IFN-γ, TNF_ < 0.001, P _IL-17A_ < 0.05)(Fig. 5A,B and G). In addition, the concentration of IL-6 was abnormally increased at the first week after syngeneic corneal transplantation, higher in comparison to the other two groups (P < 0.001), and then it gradually decreased to become similar to the allograft mice. At the third week after surgery, the concentration of IL-6 in the rejectors of the IgG1 group was significantly higher than that in the other three groups (P < 0.001) (Fig. 5E). The level of IL-2 in the syngeneic group was higher than the allogeneic group at the first week after surgery (P = 0.002), but we did not observe a statistically significant difference in IL-2 level among the groups at other time points (Fig. 5C).

### Expression of TGF-β1 mRNA in the corneal grafts

The results of TGF-β1 mRNA expression in the corneal grafts are shown in Fig. 5H. The mRNA expression of TGF-β1 was not statistically different between the syngeneic group, the anti-LAP group, and the non-rejectors of the isotype IgG1 group within 4 weeks after corneal transplantation (P > 0.05). However, compared with non-rejected allograft mice, the mRNA expression of TGF-β1 in the rejectors of the isotype IgG1 group significantly decreased at the third and fourth weeks after surgery (P_w3_ < 0.001, P_w4_ = 0.002).

## Discussion

This present study is the first to note that the percentages of CD4+LAP+ T cells and CD4+GARP+ T cells in the lymph nodes and spleens of mice after corneal transplantation are significantly lower in rejected corneas. Moreover, LAP expression in our study was consistent with GARP expression on CD4+ T cells, supporting previous studies in a mouse model suggesting that the surface LAP is also GARP-anchored in CD4+ T cells^20^. In this study, an anti-LAP mouse mAb was injected in an attempt to interfere with the expression of LAP on CD4+ T cells *in vivo*. Consequently, our findings indicate that not only the percentages of CD4+LAP+ T cells and CD4+GARP+ T cells, but also the percentages of CD4+Foxp3+ T cells significantly decreased in the lymph nodes and spleens. Previous studies found that GARP-siRNA induced not only a moderate downregulation of CD4+LAP+ T cells, but it could also partly decrease the proportion of CD4+Foxp3+ T cells^21^, because CD4+Foxp3+ T cells and TGF-β-exposed CD4+Foxp3- T cells both expressed surface LAP and GARP in mice^22^. However, some studies showed contradictory results. Da Cunha AP *et al*. found that the administration of anti-LAP resulted in a significant reduction of the number of CD4+LAP+ T cells that populate the spleen and mesenteric lymph nodes, with no effect over the CD4+Foxp3+ T cell population in experimental autoimmune encephalomyelitis model. They suggested that only a fraction of Foxp3+CD4 T cells in their model express LAP and that administration of anti-LAP does not directly influence the activity of the Foxp3+CD4 T-cells^23^.

Although the percentages of CD4+Foxp3+ T cells and CD4+LAP+ T cells were significantly decreased by anti-LAP mAb treatment in our study, the survival of corneal grafts was not affected by its administration. This could be attributed to two principal factors. First, we show that the decline of CD4+LAP+ T cells was not associated with the up-regulation of IFN-γ and IL-17A expressed on the CD4+ T cells in the secondary lymphoid organs in the anti-LAP treated mice. In the rejectors of the IgG1 treated group, however, while the percentage of CD4+LAP+ T cells was as low as in the anti-LAP group, the percentages of CD4+IFN-γ+ T cells and CD4+IL-17A+ T cells significantly increased in the lymph nodes and spleens at the third post-operative week. The importance of high IL-17 expression in the reduction of Tregs suppressor activity was further emphasized in a study of D’Ambrosio *et al*. on active ulcerative colitis patients^24^. Secondly, as Scurr *et al*. reported, Foxp3-LAP+ T cells exhibit a potent *in vitro* suppressive activity mediated by TGF-β and IL-10, and are up to 50-fold more suppressive than conventional Foxp3+ Tregs^25^. We therefore speculate that though the reduced CD4+LAP+ T cells, also expressing Foxp3, lead to a decrease in the total CD4+Foxp3+ T cell number, the residual LAP+Foxp3- T cells still had a potent enough suppressive activity and could have played an important role in the inhibition of corneal allograft rejection.

Previous studies have discussed the potential role of a high IL-10 level in the prevention of graft rejection^26,27^. Gong *et al*. demonstrated that systemic but not local application of IL-10 gene vectors prolonged corneal graft survival^28^. Our group’s previous study reported that inhibition of Th1 responses and increased concentration of CD4+IL-10+ T cells could prolong the survival of allogeneic corneal grafts in mice^29^. In this current study, the decline of CD4+IL-10+ T cells in the rejectors of the IgG1 treated mice, accompanied the down regulation of LAP expression on the third week after surgery, pointing at a possible association between the two. This may be further supported by the decrease in the percentage of CD4+IL-10+ T cells upon anti-LAP administration. This association was also noted by Abd *et al*. who stated in their study that CD4+LAP+ T cells were able to make IL-10^30^. However, in the anti-LAP treated group, the percentage of CD4+LAP+ T cells was very low, even lower than that in the rejectors of the IgG1 mice, but the corneal graft remained transparent. This may indicate that the decline of CD4+IL-10+ T cells does not play a decisive role in corneal rejection. Nevertheless, an expansion of CD4+LAP+ T cells increases the expression of CD4+IL-10+ T cells in the draining lymph nodes and spleens, which may contribute to the graft protecting effect.

In our study, despite the use of anti-LAP mAb, we did not find a change in the concentrations of IFN-γ, TNF, IL-2, IL-4, IL-6, IL-10 and IL-17A in the aqueous humor after allogeneic corneal transplantation. We did, however, find that the levels of IFN-γ, TNF, IL-6 and IL-17A were elevated during corneal rejection. Since most studies confirmed that corneal allograft rejection is thought to be a delayed-type hypersensitivity (DTH) reaction, mainly mediated by T helper (Th)1-type and T helper (Th)17-type^31^, this result is within expected lines. This is further supported by the finding that neutralization of IFN-γ promotes the emergence of Tregs and therefore result in a profound increase in graft survival^32^, and that neutralization of mouse IL-17 bioactivity with anti-IL-17 mAb improves allogeneic corneal graft survival^33^. As for IL-6, a pro-inflammatory cytokine, it was shown to induce the production of IL-17A^34^. Yin *et al*. also deemed that IL-6 might be involved in the transition to an environment where IL-17A could directly affect the viability of corneal allograft^35^.

In summary, our study indicates that CD4+LAP+ T cells help maintain the immune-privileged status of corneal allografts. Blockage of LAP with anti-LAP mAb has resulted in a decreased number of CD4+LAP+ T cells in the lymph nodes and spleens, but it did not accelerate corneal allograft rejection in a mouse model. The expression of CD4+LAP+ T cells was strongly correlated with CD4+IL-10+ T cells. However, CD4+IL-10+ T did not seem to play a key role in the process of graft rejection. Allograft rejection was mainly impacted by the percentage of CD4+IFN-γ+T cells and CD4+IL-17A+ T cells, not CD4+IL-4+ T cells in the lymph nodes and spleens. Increasing and enhancing the number and function of CD4+LAP+ T cells may have a positive impact on promoting corneal allograft survival.
